# Effect of Methionine Deficiency on the Growth Performance, Serum Amino Acids Concentrations, Gut Microbiota and Subsequent Laying Performance of Layer Chicks

**DOI:** 10.3389/fvets.2022.878107

**Published:** 2022-04-25

**Authors:** Yafei Liu, Dehua Wang, Lihong Zhao, Jianyun Zhang, Shimeng Huang, Qiugang Ma

**Affiliations:** State Key Laboratory of Animal Nutrition, College of Animal Science and Technology, China Agricultural University, Beijing, China

**Keywords:** methionine deficiency, egg-laying chicks, growth performance, amino acid, intestinal development

## Abstract

This study was conducted to investigate the effect of methionine (Met) deficiency in the rearing period on the growth performance, amino acids metabolism, intestinal development and gut microbiome of egg-laying chicks and the continuous effects on the performance, egg quality, and serum amino acids metabolism of the subsequent development process. Three hundred sixty one-day-old chicks were randomly divided into two groups and fed on a basal diet (NC group, Met 0.46%) and Met deficiency diet (Met- group, Met 0.27%). Each group included six replicates with 30 chicks per replicate. The trial lasted 6 weeks (0–6 weeks), both groups were fed the same basal diet which met the needs of Met during the observation period (7–24 weeks). Results showed that Met deficiency significantly decreased (*P* < 0.05) body weight (BW), average daily weight gain (ADG), average daily feed intake (ADFI) and tibia length (TL) compared to the NC group during the trial period (0–6 weeks). Also, Met deficiency dramatically increased (*P* < 0.05) feed conversion ratio (FCR) during the trial and observation period (7–24 weeks). In addition, during the observation period, the BW and ADG were decreased (*P* < 0.05) in the Met- group. Moreover, Met- group decreased (*P* < 0.05) villi height and villi height/crypt depth ratio in jejunum at 6th weeks. In addition, the concentrations of serum main free amino acids (FAA) in the Met- group were significantly increased (*P* < 0.05) at 6th weeks, while were decreased at 16th weeks. Based on the α-diversity and PCoA analysis in β-diversity, there were no significant differences in the cecal microbial composition between NC and Met- groups. However, the LEfSe analysis revealed that differential genera were enriched in the NC or Met- groups. The Haugh unit, shell thickness and egg production in the Met- group were significantly lower (*P* < 0.05) than in the NC group. In conclusion, these results revealed that dietary supplementation of appropriate Met could substantially improve the growth performance, host amino acid metabolism and intestinal development and continuously improve the laying performance and thus boost the health of growing hens.

## Introduction

The importance of Methionine (Met) is indicated by its significant functions in protein synthesis, methylation reaction of DNA, and as a precursor in the synthesis of cysteine, glutathione, and taurine (1–3). Met is an essential amino acid that can't be synthesized by animals, particularly in poultry. In layers, Met is classified as the first limiting amino acid. Dietary Met levels directly affect production performance and egg quality (4, 5). Met is essential for the growth, production, development of feathers and immune responses enhancement of poultry (6–8). For laying hens, an optimal dose of Met supplementation in diets can improve the efficiency of protein utilization and affect the egg weight, albumen and yolk ratio (9). However, excessive supplementation with Met impaired growth and had no significant beneficial effect on laying performance (10, 11). Previous studies showed that Met deficiency in poultry reduced growth, feed intake (FI), body weight gain (BWG), egg size, production and increased feed conversion ratio (FCR) for layers and breeders (12–14). Additionally, low methionine diets decrease laying performance and egg quality (15, 16). Moreover, a deficiency of Met in the diet of laying hens impairs the whole-body protein metabolism, significantly reducing protein synthesis (17). In addition, Met has been generally recognized as a critical molecule in gut microbial metabolism. Met deficiency also alters the gut microbial structure (18). Met deficiency induces the small intestinal villus atrophy, decreases goblet cell number and diminishes small intestinal redox capacity affecting intestinal development (19). The small intestine is one of the most important organs responsible for the digestion and absorption of nutrients required for growth and development; meanwhile, as the primary media of feed digestion and absorption, the activities of digestive enzymes are inextricably linked with the digestion and utilization of nutrients (20).

The rearing period is critical for the growth and development of poultry. During the rearing period, the nutritional requirements for layer chicks should be ensured so that the chicks can grow rapidly and healthy and provide a solid foundation for later growth, development, and production. However, no research has been conducted on the investigation of methionine deficiency in the serum amino acids concentration, gut microbiota, and intestinal development of egg-laying chicks. Meanwhile, the impact of Met deficiency during the rearing period on the subsequent development of egg-laying chicks has not been reported yet. Therefore, the objective of the present experiment was to determine the effects of Met deficiency in the rearing period diets on growth performance, intestinal development, gut microbiota, the content of serum free amino acids of egg-laying chicks and evaluate its continuous impacts on the subsequent development index and production performance.

## Materials and Methods

### Birds, Diets and Management

All experimental procedures of the present study were permitted by the China Agricultural University Animal Care and Use Committee (AW13301202-1-11, Beijing, China).

A total of 360 one-day-old Peking Pink strain egg-laying chicks (Yukou Poultry Co., Ltd. of Beijing, China) were randomly divided into two groups. The dietary groups were as follows: NC group (NC), fed basal diet, Met 0.46%; Met- group (Met-), fed Met deficiency diet, Met- 0.26%. Each group included six replicates with 30 chicks per replicate. The trial lasted 6 weeks. Subsequently, the same diet was fed to carry out a continuous observation of the test chicks until the laying period (7–24 weeks). Birds were housed in stainless steel cages (W 65 × L 62 × H 37 cm). Room temperature and artificial light were controlled, and the vaccination programs were implemented according to the management guide of Peking Pink hens. Birds had *ad libitum* access to water throughout the experimental period. Chicks were fed *ad libitum* commercial corn- and soybean meal-based diet containing the nutritional requirements for layer chicks (National Research Council, 1994) (Table 1).

**Table 1:** Ingredients and nutrient content of the diets (% DM).

**Ingredients (%)**	**0–6 weeks**		**7–12 weeks**	**13–16 weeks**	**17–20 weeks**	**21–24 weeks**
	**NC**	**Met-**				
Corn	68.20	68.20	67.18	67.26	66.47	65.05
Dehulled soybean meal	25.70	25.70	23.50	19.00	22.80	24.20
Zeolite powder	0.00	0.19	–	–	–	-
Wheat bran	–	–	5.60	9.50	3.60	0.00
Calcium monohydrogen phosphate	2.00	2.00	1.50	1.80	1.70	1.70
Limestone	1.30	1.30	1.20	1.40	4.60	8.20
NaCl (salt)	0.30	0.30	0.30	0.30	0.30	0.30
Vitamin premix^a^	0.04	0.04	0.04	0.03	0.03	0.03
Mineral premix^b^	0.30	0.30	0.30	0.30	0.30	0.30
50%Choline chloride	0.10	0.10	0.10	0.10	0.10	0.10
L-Lysine-HCl (78.5%)	0.15	0.15	0.07	0.05	0.00	0.00
DL-Methionine	0.19	0.00	0.16	0.16	0.10	0.12
Threonine	0.07	0.07	–	–	–	-
Tryptophan	0.02	0.02	–	–	–	-
Isoleucine	0.08	0.08	–	–	–	-
Alanine	1.55	1.55	–	–	–	-
Total	100	100	100	100	100	100
Nutrient (%)^c^						
Crude protein	19.06	19.06	16.51	15.05	15.52	16.04
AME (MJ/kg)	2.85	2.85	2.80	2.74	2.70	2.69
Ca	1.02	1.02	0.89	1.01	2.20	3.60
Total P	0.67	0.67	0.64	0.69	0.66	0.65
Non-phytate phosphorus	0.45	0.45	0.39	0.44	0.39	0.39
Methionine	0.46	0.27	0.40	0.40	0.35	0.38
TSAA	0.74	0.55	0.66	0.67	0.62	0.65
Lys	1.01	1.01	0.85	0.75	0.75	0.78
Trp	0.22	0.22	0.17	0.17	0.15	0.16
Thr	0.72	0.72	0.66	0.63	0.57	0.59
Ile	0.76	0.76	–	–	–	-

a *Vitamin premix supplied (per kg of diet): vitamin A, 11,700 IU(0–6 weeks); vitamin A, 8,000 IU;(7–24 weeks);vitamin D3, 3,600 IU; vitamin E, 21 IU; vitamin K3, 4.2 mg; vitamin B1, 3 mg; vitamin B2, 10.2 mg; folic acid, 0.9 mg; calcium pantothenate, 15 mg; niacin 45 mg; vitamin B6, 5.4 mg; vitamin B12, 24 μg; and biotin: 0.15 mg. 7–24 weeks: vitamin A, 8,000 IU; vitamin D3, 3,600 IU; vitamin E, 21 IU; vitamin K3, 4.2 mg; vitamin B1, 3 mg; vitamin B2, 10.2 mg; folic acid, 0.9 mg; calcium pantothenate, 15 mg; niacin 45 mg; vitamin B6, 5.4 mg; vitamin B12, 24 μg; and biotin: 0.15 mg*.

b *Mineral premix provided (per kg of diet): 0–6 weeks: Cu, 6.8 mg; Fe, 66 mg; Zn, 83 mg; Mn, 80 mg; I, 1 mg*.

c *The nutrient levels were calculated values*.

### Growth Performance

The body weight and feed intake of chicks in each replicate were measured weekly. Average daily weight gain (**ADG**), average daily feed intake (**ADFI**), and feed conversion ratio (**FCR**) were calculated at different stages (0–2 weeks, 3–4 weeks, 5–6 weeks, 0–6 weeks, 7–16 weeks, and 17–24 weeks). The ADFI and FCR were corrected for mortality. Body weight (**BW**) and tibial length (**TL**) were observed until the end of the 24th week. During the feeding trial, the data was measured every two weeks to observe the influence of the rearing period on growth performance.

### Laying Performance and Egg Quality

During the observation period, eggs, broken and shell-less eggs, were collected daily; eggs were weighed and recorded daily by replicate. The see egg age, which represents the age of the first egg appearance, was recorded. Egg production and daily egg mass were calculated on a per replicate basis. The age of the first production day was calculated according to the period when the daily egg production rate reached 50% by replicate.

At weeks 21 and 24, egg quality parameters were measured based on five eggs collected at random from each replicate. The eggshell strength was measured using the egg force reader (F0241, Orka Technology Ltd) and the digital egg tester (ESTG-01, Orka Technology Ltd) was used to measure the eggshell thickness. Haugh unit and yolks color were measured using a multifunctional egg quality tester (EA-01, Orka Technology Ltd). The eggshell was weighed, yolks were separated using a separator. They were weighed on digital technical balance with a precision of ± 0.1 g to determine the relative albumen and yolk proportion.

### Blood Collection and Analysis

At the end of the 6th, 16th, and 24th weeks of the experiment, two birds were selected randomly from each replicate (12 birds per group) to collect 6 mL of blood from the wing vein. The blood was centrifuged at 3,000 rpm/min for 15 min and stored at −20 °C. After that, 0.5 mL serum was collected by pipette into tubes, added 1.5 mL 4% sulfosalicylic acid, shook at high speed for 1 min, put the samples in an ice-bath for 20 min; then added 175 μL LiOH solution (2 mol/L). After thoroughly mixed, samples were centrifuged at 12,000 rpm/min for 40 min; the supernatant was used across the 0.22 μm membrane and adjusted to the appropriate concentration. The L-8,900 amino acid analyzer (Hitachi, Japan) was used to determine the content of 17 kinds of free amino acids in the serum.

### Digestive Enzyme Activity Assay

At the end of the trial, two birds per replicate (12 birds per group) were randomly selected for euthanasia with cervical dislocation. The samples in duodenal chyme were collected and stored at −80 °C. The levels of amylase, lipase, chymotrypsin and trypsin activities in duodenum samples were detected with the corresponding kits provided by Nanjing Jiancheng Institute of Bioengineering following the manufacturer's instructions.

### Small Intestinal Morphology

Duodenum, jejunum, and ileum segments were fixed in 4% paraformaldehyde (pH 7.2) solution for 24 h, dehydrated and embedded in paraffin. Each selected sample was sectioned into six μm thickness with a microtome. The sections of the intestine were stained with hematoxylin and eosin. Using an image processing system composed of CMOS (OLYMPUS, Japan) and its special software (Imaging Technology, USA), the computer obtains the villus height, crypt depth, and ratios. Villus height and crypt depth were measured at × 40 magnification using a microscope (BA400Digital, Mike Audi Industrial Group Co., Ltd. Xiamen, China). The images were evaluated by using an Axioplan 2 microscope (Carl Zeiss, Thornwood, NY) interfaced with an Axiocam HR digital camera.

### DNA Extraction, Amplification, and Sequencing of Gut Microbiota

Cecal samples were collected and stored at −80°C. Total DNA of the cecum contents was extracted using the QIAamp Fast DNA Stool Mini Kit (Qiagen, Hilden, Germany) according to the manufacturer's instructions. To assess the quantity and purity of the DNA, the extracted DNA was determined by a NanoDrop 2000 UV-vis spectrophotometer (NanoDrop Technologies, Wilmington, DE, USA). The V3-V4 region of the 16S rRNA gene was amplified with primer pairs 338F (5′ACTCCTACGGGAGCA-3′) and 806R (5′- GGACTACHVGGGTWTCTAAT-3′) by an ABI GeneAmp^®^ 9700 PCR thermocycler (ABI, CA, USA). The PCR product was extracted from 2% agarose gel and purified using the AxyPrep DNA Gel Extraction Kit (Axygen Biosciences, Union City, CA, USA). Paired-end reads were generated with Illumina MiSeq PE250 (Shanghai MajorBio Biopharma Technology Co., Ltd., China), and the reads were filtered out with default parameters.

### Data Statistics and Analysis

Tabulated results were expressed as means with SEM. The comparison of NC group with Met- group values were performed with two-tailed Student's test for unpaired data. All analyses were performed with SPSS 21 software (SPSS, Inc., Chicago, IL, USA). Statistical significance was assigned at *P* < 0.05.

The raw paired-end reads were assembled into longer sequences, and the PANDAseq (version 2.9) was used to filter the quality and remove the low-quality reads. The high-quality sequences were clustered into operational taxonomic units (OTUs) based on a 97% sequence similarity using UPARSE (version 7.0) in QIIME (version 1.17) and using UCHIME to remove the chimeric sequences, and taxonomy was assigned to OTUs using the RDP classifier (21). UPARSE (version 7.0) was used to cluster the subsequent clean reads and annotated with the SILVA 16S rRNA gene database using the MOTHUR program (version v.1.30.1). Alpha-diversity (the Sob index, Chao index, Shannon index, and Ace index) was calculated based on the profiles of OTU by the MOTHUR program. Bar plots and heat maps were generated with the “vegan” package in R (version 3.3.1). A Venn diagram was generated to visualize the shared and unique OTUs among groups using the R package “Venn Diagram” based on the occurrence of OTUs across groups regardless of their relative abundance. principal coordinate analysis (PCoA) was performed based on the Bray-Curtis distance using QIIME (version 1.17). An analysis of similarities (ANOSIM) was performed to compare the similarity of bacterial communities between groups using the “vegan” package of R (version 3.3.1). The LDA effect size (LEfSe) analysis was performed to identify the bacterial taxa differentially enriched in different bacterial communities.

## Results

### Effects of Met Deficiency on Growth Performance

As shown in Table 2, Met deficiency (only 6 weeks for laying chicks) significantly decreased the BW and TL on weeks 2, 4 and 6 (*P* < 0.05). Subsequently, during the observation period (from week seven to week 24), BW was significantly reduced (*P* < 0.05) at 16 weeks. Compared with the NC group, ADFI and ADG of Met- group were decreased on the trial and observation period (*P* < 0.05). On weeks of 7–16, ADFI of Met- group was significantly increased, but ADG of the Met- group was significantly decreased (*P* < 0.05). FCR was significantly higher in Met- group (*P* < 0.05) of the whole test period and the 7–16 weeks of the observation period.

**Table 2:** Effects of Met deficiency in rearing period diets on growth performance of egg-laying chicks.

**Period**	**Group** ^**1**^				
	**NC**	**Met-**	**Difference^**c**^**	**SEM^**2**^**	***P value***
	BW (g)				
2 wk	121.02^a^	114.18^b^	−6.83	1.06	<0.001
4 wk	250.01^a^	224.73^b^	−25.28	3.89	<0.001
6 wk	419.45^a^	360.78^b^	−58.67	8.97	<0.001
16 wk	1,209.75^a^	1,177.41^b^	−32.34	6.91	0.022
18 wk	1,302.05	1,271.41	−30.64	7.59	0.052
24 wk	1,533.05	1,524.89	−9.08	8.26	0.611
	TL (mm)				
2 wk	45.26^a^	44.53^b^	−0.72	0.16	0.013
4 wk	58.24^a^	56.68^b^	−1.56	0.30	0.003
6 wk	72.38^a^	69.12^b^	−3.26	0.57	<0.001
16 wk	100.01	100.01	−0.01	0.30	0.994
18 wk	99.24	99.82	0.58	0.41	0.510
24 wk	99.77	99.99	0.23	0.59	0.858
	ADFI (g/bird/d)				
0–2 wk	11.80	11.87	0.07	0.05	0.506
3–4 wk	21.25^a^	20.27^b^	−0.98	0.17	<0.001
5–6 wk	30.95^a^	28.14^b^	−2.81	0.47	<0.001
0–6 wk	21.58^a^	20.30^b^	−1.28	0.21	<0.001
7–16 wk	54.06^b^	54.52^a^	0.45	0.11	0.030
17–24 wk	85.39	85.44	0.05	0.17	0.889
	ADG (g/bird/d)				
0–2 wk	6.19^a^	5.67^b^	−0.53	0.08	<0.001
3–4 wk	9.92^a^	8.50^b^	−1.42	0.23	<0.001
5–6 wk	12.10^a^	9.72^b^	−2.39	0.38	<0.001
0–6 wk	9.48^a^	8.01^b^	−1.47	0.22	<0.001
7–16 wk	12.31^a^	11.82^b^	−0.49	0.11	0.022
17–24 wk	5.80^b^	6.21^a^	0.42	0.11	0.048
	FCR				
0–2 wk	1.91^b^	2.10^a^	0.19	0.03	<0.001
3–4 wk	2.14^b^	2.38^a^	0.24	0.04	<0.001
5–6 wk	2.56^b^	2.90^a^	0.34	0.05	<0.001
0–6 wk	2.28^b^	2.54^a^	0.26	0.04	<0.001
7–16 wk	4.40^b^	4.61^a^	0.22	0.04	0.003
17–24 wk	14.78	13.78	−1.00	0.26	0.052

a, b *Means with different superscripts within a row differ significantly (P < 0.05)*.

c *Means difference of mean value between NC and Met- group*.

1 *NC, fed basal diet, Met 0.46%; Met-, fed Met deficiency diet, Met- 0.26%*.

2 *SEM: standard error of the means*.

*TL, tibial length*.

### Effects of Met Deficiency on Small Intestinal Digestive Enzyme Activity

Met deficiency had no significant effects (*P* > 0.05) on the activity of amylase, lipase, trypsin, and chymotrypsin (Table 3).

**Table 3:** Effects of Met deficiency in rearing period diets on digestive enzymes activities in duodenum of egg-laying chicks at 6 weeks.

	**Group** ^**1**^				
**Item**	**NC**	**Met-**	**Difference^**a**^**	**SEM^**2**^**	***P* value**
Typsin activity (U/mg prot)	591.03	388.59	−202.44	150.86	0.563
Chymotrypsin activity (U/mg prot)	2.15	2.93	0.79	0.58	0.557
Lipase activity (U/mg prot)	4.69	4.05	−0.64	1.01	0.787
Amylase activity (U/mg prot)	0.13	0.12	−0.01	0.01	0.588

a *Means difference of mean value between NC and Met- group*.

1 *NC, fed basal diet, Met 0.46%; Met-, fed Met deficiency diet, Met- 0.26%*.

2 *SEM: standard error of the means*.

### Effects of Met Deficiency on Laying Performance and Egg Quality

As shown in Table 4, Met deficiency significantly affected the laying performance of hens. For example, main parameters, such as egg production, average egg weight and egg mass in the Met- group were significantly lower (*P* < 0.05) than that in the NC group in 18–24 weeks. Also, the age of the first production day was significantly postponed (*P* < 0.05) in the Met- group. Met deficiency especially increased the unqualified egg rate (*P* < 0.05) in 18–24 weeks.

**Table 4:** Effects of Met deficiency in rearing period diets on Subsequent laying performance of laying hens.

	**Group** ^**1**^				
**Item**	**NC**	**Met-**	**Difference^**c**^**	**SEM^**2**^**	***P* value**
See egg age (d)	130.00	134.50	4.50	1.367	0.100
The age of the first production day (d)	142.83^b^	144.83^a^	2.00	0.405	0.006
	18–20 weeks				
Egg production (%)	10.78^a^	6.71^b^	−4.07	0.009	0.006
Average egg weight (g)	42.64^a^	41.26^b^	−1.37	0.265	0.006
Egg mass (g/bird/d)	4.61^a^	2.78^b^	−1.83	0.366	0.005
	21–22 weeks				
Egg production (%)	75.92^a^	62.30^b^	−13.62	0.024	<0.001
Average egg weight (g)	48.24	46.86	−1.38	0.368	0.057
Egg mass (g/bird/d)	36.96^a^	29.96^b^	−7.00	1.170	<0.001
	23–24 weeks				
Egg production (%)	97.25	96.64	−0.61	0.003	0.380
Average egg weight (g)	53.33	52.69	−0.64	0.212	0.139
Egg mass (g/bird/d)	52.08	51.02	−1.07	0.325	0.102
	18–24weeks				
Egg production (%)	61.32^a^	55.21^b^	−6.10	0.011	0.001
Average egg weight (g)	48.07^a^	46.94^b^	−1.13	0.227	0.005
Egg mass (g/bird/d)	31.21^a^	27.92^b^	−3.30	0.551	<0.001
Unqualified egg rate (%)	0.09	0.26	0.16	0.000	0.072

a, b *Means with different superscripts within a row differ significantly (P < 0.05)*.

c *Means difference of mean value between NC and Met- group*.

1 *NC, fed basal diet, Met 0.46%; Met-, fed Met deficiency diet, Met- 0.26%*.

2 *SEM: standard error of the means*.

As shown in Table 5, the Haugh unit in the Met- group was significantly lower (*P* < 0.05) than that in the NC group at 21 weeks. Meanwhile, the eggshell percentage and thickness in the Met- group were significantly lower (*P* < 0.05) than that in the NC group in 24 weeks.

**Table 5:** Effects of Met deficiency in rearing period diets on egg quality of 21 and 24 weeks laying hens.

	**Group** ^**1**^				
**Item**	**NC**	**Met-**	**Difference^**c**^**	**SEM^**2**^**	***P* value**
**21 weeks**
Egg shape index	1.31	1.30	−0.01	0.006	0.291
Shell percentage (%)	9.52	9.38	−0.14	0.097	0.485
Albumen percentage (%)	68.85	68.79	−0.06	0.268	0.914
Yolk percentage (%)	21.63	21.83	0.20	0.249	0.695
Shell color	57.28	54.69	−2.59	0.903	0.155
Yolk color	4.17	4.17	0.00	0.102	1.000
Shell strength (kg/cm^2^)	4.32	4.07	−0.25	0.125	0.333
Shell thickness (mm)	0.401	0.394	−0.01	0.004	0.413
Haugh unit	82.94^a^	73.96^b^	−8.99	2.135	0.033
**24 weeks**
Egg shape index	1.30	1.30	0.00	0.005	0.866
Shell percentage (%)	11.10^a^	10.48^b^	−0.62	0.123	0.009
Albumen percentage (%)^3^	64.61	65.29	0.67	0.299	0.266
Yolk percentage (%)^3^	24.29	24.24	−0.06	0.273	0.921
Shell color	60.20	58.58	−1.62	1.156	0.491
Yolk color	4.22	4.39	0.17	0.104	0.431
Shell strength (kg/cm^2^)	4.17	3.89	−0.28	0.078	0.074
Shell thickness (mm)	0.408^a^	0.392^b^	−0.02	0.004	0.024
Haugh unit	83.92	82.76	−1.16	1.274	0.657

a, b *Means with different superscripts within a row differ significantly (P < 0.05)*.

c *Means difference of mean value between NC and Met- group*.

1 *NC, fed basal diet, Met 0.46%; Met-, fed Met deficiency diet, Met- 0.26%*.

2 *SEM: standard error of the means*.

3 *The albumen and yolk percentage were calculated based on the whole egg weight*.

### Effects of Met Deficiency on Small Intestinal Morphology

**Figure 1:**
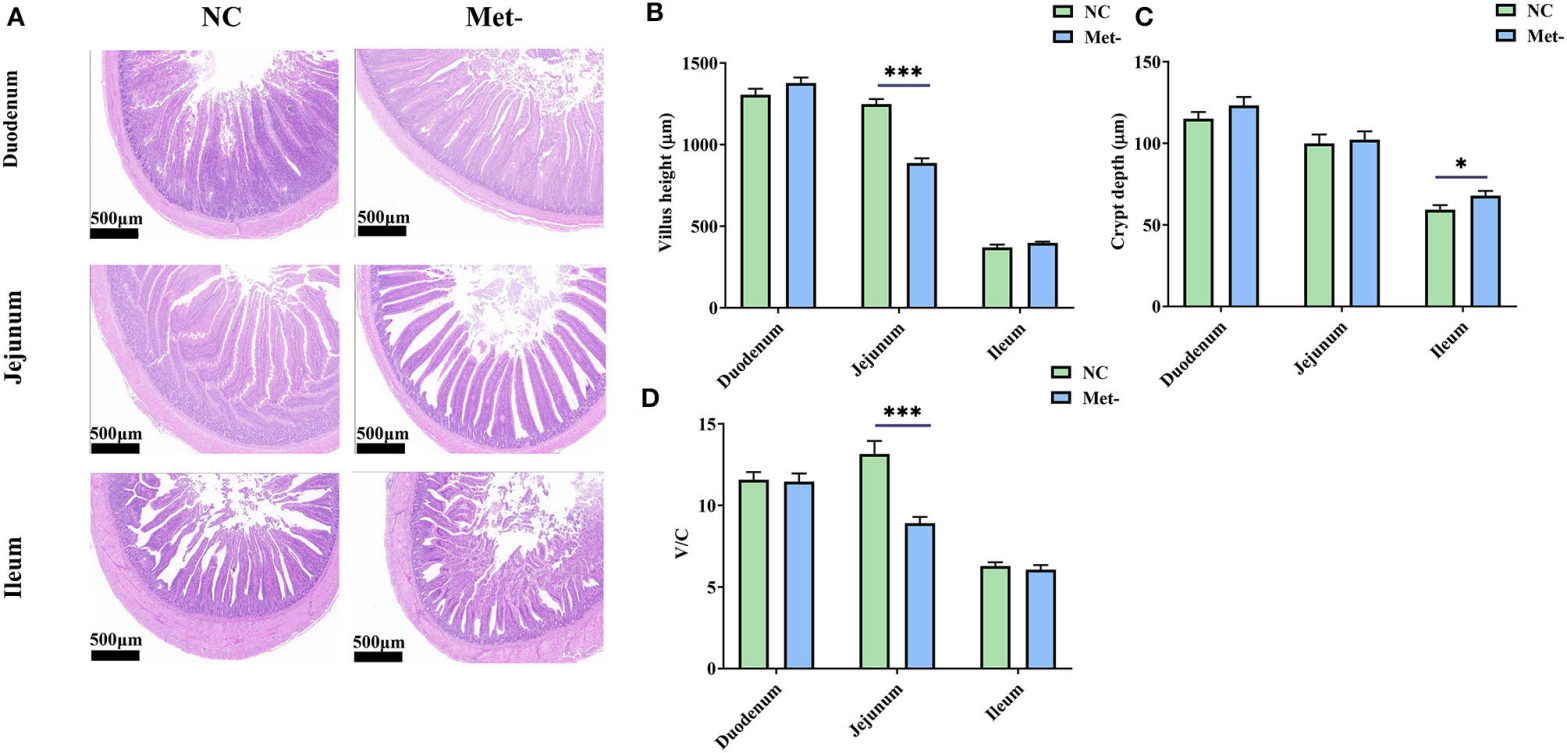
Effects of Met deficiency in rearing period diets on small intestine morphologic structure of egg-laying chicks. **(A)** Representative samples from histological stained sections showed Met- group had lower jejunal villus height and deeper ileal crypt depth. **(B)** Villus height of duodenum, jejunum, and ileum. **(C)** Crypt depth of duodenum, jejunum, and ileum. **(D)** Villus height-to-crypt depth ratio (V/C) of duodenum, jejunum, and ileum. Data were presented as means ± SEM. Significant differences were tested by student's *t*-test. *Indicates significant difference, *P* < 0.05; ***, *P* < 0.001.

Chicks treated with Met deficiency diet significantly decreased the height of jejunal villi (Figures 1A,B) and the V/C value (*P* < 0.05) (Figure 1D) and significantly increased the depth of ileum crypts (*P* < 0.05) (Figure 1C).

### Effects of Met Deficiency on Content of Serum Free Amino Acids

**Figure 2:**
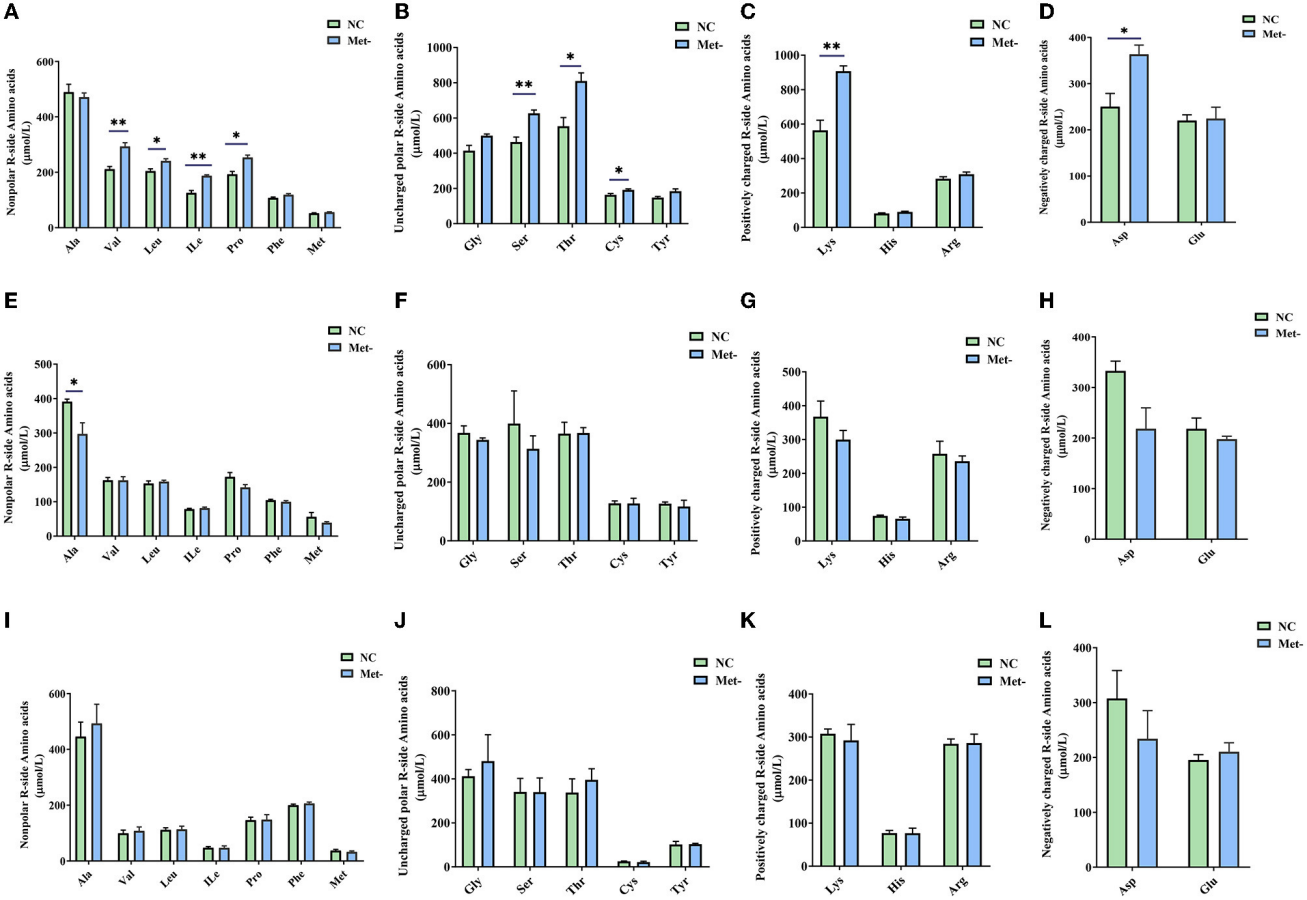
Effects of Met deficiency in rearing period diets on serum concentration of amino acids (AA) of laying hens. **(A–D)** serum concentration of amino acids (AA) of laying hens at 6th weeks, **(A)** Nonpolar R-side Amino acids. **(B)** Uncharged polar R-side Amino acids. **(C)** Positively charged R-side Amino acids. **(D)** Negatively charged R-side Amino acids. **(E–H)** serum concentration of amino acids (AA) of laying hens at 16th weeks. **(E)** Nonpolar R-side Amino acids. **(F)** Uncharged polar R-side Amino acids. **(G)** Positively charged R-side Amino acids. **(H)** Negatively charged R-side Amino acids. **(I–L)** serum concentration of amino acids (AA) of laying hens at 24th weeks. **(I)** Nonpolar R-side Amino acids. **(J)** Uncharged polar R-side Amino acids. **(K)** Positively charged R-side Amino acids. **(L)** Negatively charged R-side Amino acids. Data were presented as means ± SEM. Significant differences were tested by student's *t*-test. *Indicates significant difference, *P* < 0.05; **, *P* < 0.01.

Met deficiency significantly affected the content of amino acids in the serum of egg-laying chicks. The content of Thr, Ser, Cys, Val, Ile, Leu, Pro, Asp, and Lys in the Met- group on the 6th week was higher (*P* < 0.05) than that in the NC group (Figures 2A–D). Moreover, the content of Ala in the NC group was higher (*P* < 0.05) than that in the Met- group on the 16th week (Figure 2E). However, there were no significant differences (*P* > 0.05) between the two groups in the content of serum free amino acids on the 24th week (Figures 2I–L).

### Effects of Met Deficiency on the Cecal Microbiota

**Figure 3:**
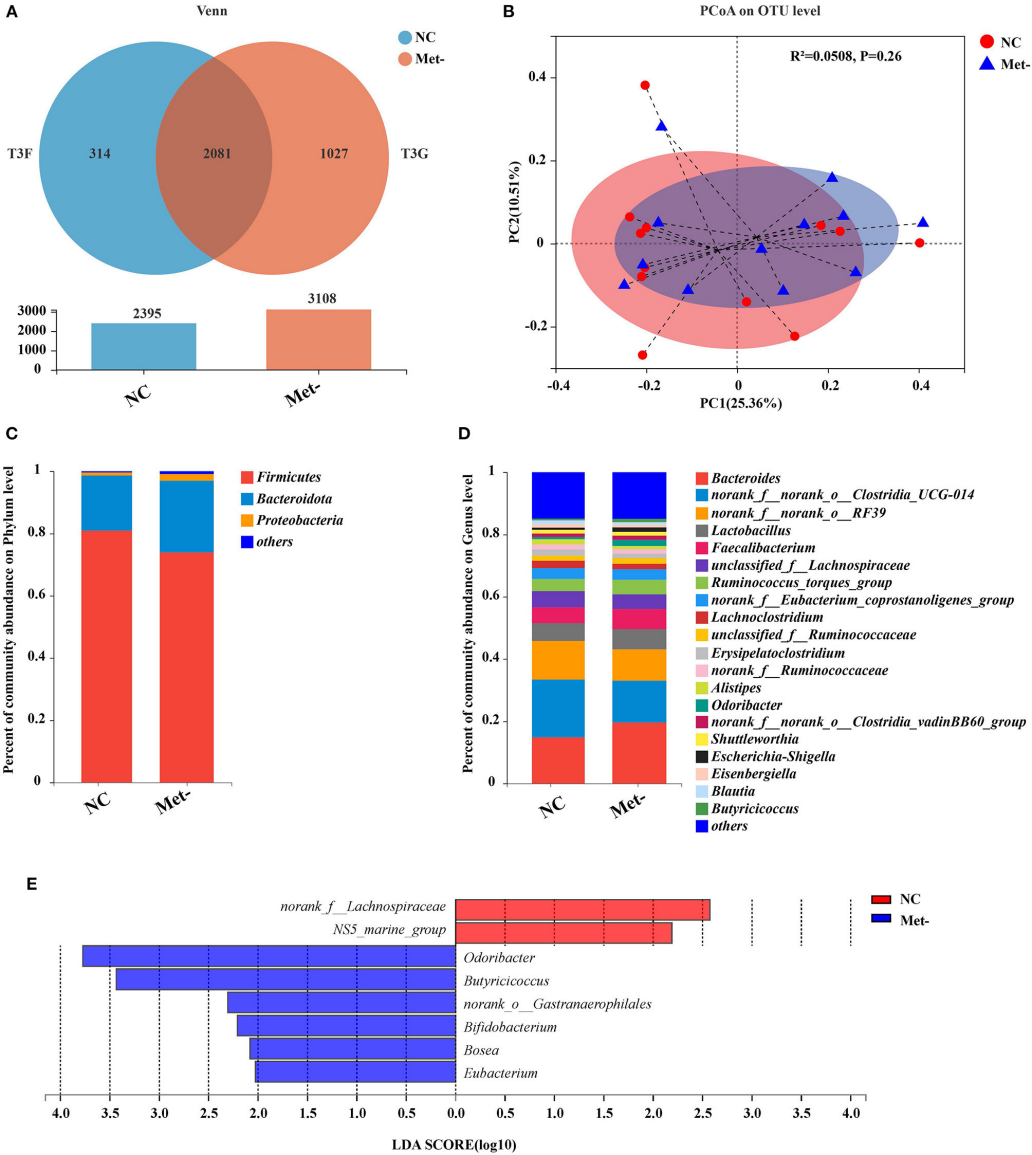
Effects of Met deficiency in rearing period diets on the gut microbiota of egg-laying chicks at 6 weeks. **(A)** Venn diagram of the shared and total operational taxonomic units (OTUs) in the NC and Met- groups. **(B)** The principal coordinate analysis (PCoA) (Bray-Curtis distance) plot of the gut microbial community structure between the NC and Met- group. **(C)** Relative abundance of gut microbiota at the phylum level. **(D)** Relative abundance of gut microbiota at the genus level. **(E)** Histograms of the linear discriminate analysis (LDA) score (threshold ≥ 2) in Met- and NC groups are plotted. Linear discriminate analysis effect size (LEfSe) was performed to determine the difference in abundance.

16S rDNA sequencing was performed to investigate how Met deficiency impacted the gut microbiota composition of egg-laying chicks. Based on the Venn diagram illustrating the overlap of bacterial OTUs, the NC group had a total of 2,395 OTUs, and the Met- group, had 3,108 OTUs. A total of 2,081 common OTUs were shared between the two groups. The NC group exhibited the number of unique sequences (314 OTUs) and the total number of unique sequences in the Met- group was 1,027 OTUs (Figure 3A).

The alpha-indices (Sobs index, Ace index, Chao index and Shannon index) were used to describe the degree of cecal microbial diversity. As shown in Supplementary Figure 1, it was found that no difference was observed in the Shannon index between the two groups (*P* > 0.05). Meanwhile, there was no significant difference in other indices between the group (*P* > 0.05). The relative abundance at phylum and genus levels was studied. Principal coordinate analysis (PCoA), a multivariate statistical analysis method suitable for high-dimensional data was performed (Figure 3B). At the phylum level, Bacteroidetes and Firmicutes were the two major bacteria in the cecum of chicks, accounting for more than 90% (NC group, 98.62%; Met- group, 97.03%) of the cecum bacterial community (Figure 3C). Additionally, the Met- group had lower Firmicute/ Bacteroidetes ratios and higher Bacteroidetes than the NC group (*P* > 0.05). At the genus level, the most common genera in the NC and Met- groups were accounted by *Bacteroides*, norank_f_norank_o_*Clostridia*_UCG014, norank_f_norank_o_RF39, *Lactobacillus, Faecalibacterium* and Unclassified_ f_ *lachnospiraceue* (Figure 3D). The level of predominant genera such as *Bacteroides, Lactobacillus* and *Faecalibacterium* in the Met- group was higher than the NC group. The relative abundance of *Bacteroides* in the NC group was 14.85%, and that in the Met- group was 19.62%. The data showed the composition of the gut microbiota in the Met- group was not significantly altered. To further determine which bacterial taxa contributed to the differences both statistically and biologically, we utilized linear discriminant analysis (LDA) effect size (LEfSe) analysis (Figure 3E). As shown in Figure 3E, a variety of genera were significantly enriched in the Met- group compared to the NC group, including *Odoribacter, Butyricicoccus*, norank_o__*Gastranaerophilales, Bifidobacterium, Bosea* and *Eubacterium*.

## Discussion

As Met is usually the first or second limiting amino acid (22), its adequate supply of Met is essential in farm animal diets. Met deficiency had different effects on animals, such as reduced protein synthesis and inhibited growth development (23, 24). For poultry, Met deficiency decreased the egg quality and laying performance, such as lowering shell thickness and egg weight (13, 15, 25). Our results demonstrated that the methionine-restricted diet containing 0.26% Met significantly affected BW and TL of egg-laying chicks during the trial period (0–6 weeks). Compared with the NC group (Met 0.46%), the ADFI and ADG of Met- group (Met 0.26%) were significantly decreased during the observation period (from week seven to week 24). The result agrees with the previous studies that found that Met deficiency had significantly decreased the growth performance, such as decreased BW, FI, and increased FCR (26–28). These results showed that Met deficiency (Met 0.26%) affected the growth performance of egg-laying chicks during the rearing period and had the subsequent effect on the growth performance: BW and ADG of the Met- group were significantly decreased, but FCR was increased.

Egg quality is a significant concern for consumers and egg producers alike. The adverse effects of Met deficiency during the laying period on egg quality, such as the decreased Haugh unit and the lower shell thickness, had been widely reported (29, 30). Surprisingly, the present study showed chicks who fed Met deficient diet during the rearing period (0–6weeks) significantly reduced egg quality; even normal diets of 18 weeks could not alleviate the adverse effects of Met deficiency during the rearing period. It indicated that the effect of dietary methionine deficiency during the rearing period on chicks is sustainable. As we know that the life span of commercial laying hens is close to 80 weeks. Although we used the observation period (7–24 weeks) to study whether Met deficiency during the rearing period will have a lasting impact on growth performance and egg quality of laying hens, but the further work is needed to evaluate the duration of these effects, because the observation period of this study is relatively short for the life span of commercial laying hens.

It is plausible that dietary deficiency of an essential amino acid could affect digestibility, transport and absorption of nutrients. But our results showed that Met deficiency did not have significant effect on the digestive enzyme activity. These results agree with study by Nitsan et al., who stated that the enzymic activities (trypsin, chymotrypsin, and amylase) were scarcely affected by the level of Met of chicks (31).

The serum concentration of AA results from the amount and form of ingested AAs, which reflects AA absorption (32). Met takes part in many critical metabolic pathways, including protein synthesis, cysteine and carnitine metabolism and one-carbon metabolism (33). To a certain extent, the concentration of free amino acids in serum can reflect the metabolism of amino acids. In this study, the higher levels of Ser and Cys in the Met- group were observed at week 6. Met and Cys are the key factors of one-carbon metabolism, and Ser and Gly are the main sources of one-carbon groups (34, 35). The requirement for Ser is reduced when both Met and Cys are supplied adequately in broilers. When the Met was supplied insufficiently, the levels of Ser and Cys may be altered (36). At week 16, the lower content of Ala in Met- group was observed, which was consistent with the study by Wan et al. (37). They found that plasma Ala concentration was linearly increased as DL-Met supplementation level increased. In this study, these results clearly demonstrated that Met deficiency had a significant influence on the content of amino acids in serum of chicks. However, how the Met deficiency affects the serum AA concentration remains to be elucidated.

Met is critical for a rapidly growing animal (38). Met plays a vital role in intestinal development and maintains the integrity of the intestinal mucosa and barrier function (39). Notably, when comparing the EAA that is being metabolized in the gastrointestinal tract, on average, the utilization of Met tends to be greater than other EAA. Therefore, there appears to be a specific functional need for Met in the gastrointestinal tract of animals. In this study, the current study revealed the villi height of jejunal was decreased in the Met- group. Conversely, the crypts depth of the ileum was increased. Zhang et al. found that dietary Met supplementation increased villus height of the ileal, which improved the small intestinal morphology of Pekin ducks (40). Shen et al. (41) found that the supplementation of Met could increase the villus height and decrease the crypts depth of young chickens. On the contrary, Met deficiency caused the decrease of villus height and the increase of crypts depth. The amino acid imbalance caused by the lack of methionine during the rearing period may also be one of the reasons that hinder intestinal development besides.

Gut microbial communities play critical roles in animal health and productivity. There is increasing evidence that microbes in the gastrointestinal tract may play an important role in AA metabolism and host protein (42). Notably, Dai et al. discovered that AA could regulate the composition of the intestinal bacterial population from the small intestine in pigs (43). In this study, we found that there were no significant differences in alpha diversity indices between NC (Met 0.46%) and Met- group (Met 0.26%), which was similar to the previous study (44). Wu et al. found that D-methionine supplementation had no effects on alpha diversity indices in male Wistar rats (176–200 g, 6 weeks old), who uncovered the Met diet does not alter gut microbiota structure (45). Meanwhile, Wallis et al. found that when sexes were combined, there were no differences in the composition of the intestinal microflora between the control and Met restriction groups (18). Interestingly, *Firmicutes* and *Bacteroidetes* were the major phyla in the cecal digesta of chickens. Numerous studies have consistently demonstrated that the *Firmicutes*/*Bacteroidetes* (F/B) proportion is increased in obese people compared to lean people and tend to decrease with weight loss (46). Koliada et al. (47) found that the content of *Firmicutes* was gradually increased while the relative abundance of *Bacteroidetes* was decreased with increasing body mass index in Ukrainian population. In the present study, at the phylum level, the Met- group (Met 0.26%) had lower *Firmicute*/*Bacteroidetes* ratios and higher *Bacteroidetes* than the NC group (Met 0.46%), which may support our results that the BW in the Met- group was decreased. *Eubacterium* is linked with amino acid fermentation (48). These results showed that the genera of *Eubacterium* were decreased in the Met-group compared to the NC group.

*Lactobacillus* has been extensively studied and identified as the predominant amino acid-fermenting bacteria (49, 50). In this study, the results showed that the genera of *Lactobacillus* were higher in the Met-group compared to the NC group. The alteration of *Eubacterium* and *Lactobacillus* was associated with the Amino acid metabolism pathway. Recent research has demonstrated that Met can regulate animal's metabolic processes and digestive functioning (51). Meanwhile, Met is the main source of butyrate (48). However, the interactions between the gut microbiota and Met deficiencies are still unclear, and more research studies are needed.

## Conclusions

According to the results in this study, Met deficiency during the trial period (0–6 weeks) reduced growth performance, changed the concentration of serum amino acids, hindered intestinal development, affected the gut microbial community and structure of layer chicks. Met deficiency in the rearing period induced the imbalance of serum amino acids and affected the current growth performance and had a continuously adverse impact on the growth and production performance of egg-laying chicks during the observation period (from week 7–24). Therefore, it's critical to meet the needs of Met in the rearing period for subsequent growth development and production performance of egg-laying chicks.

## Data Availability Statement

The datasets presented in this study can be found in online repositories. The names of the repository/repositories and accession number(s) can be found below: https://www.ncbi.nlm.nih.gov/, PRJNA791195.

## Ethics Statement

The animal study was reviewed and approved by China Agricultural University Animal Care and Use Committee.

## Author Contributions

DW and QM designed the study. DW conducted the experiments. DW and YL collected and analyzed the samples. YL, DW, and SH guided to analyze the experimental data. YL drafted the manuscript. SH, LZ, JZ, and QM polished the manuscript and finished the submission. YL, DW, SH, LZ, JZ, and QM helped with revisiting and reviewing the manuscript. QM obtained funds and managed project. All authors read and approved the final manuscript.

## Funding

This study was supported by a special fund for China Agricultural Research System program (Grant No. CARS-40-K08).

## Conflict of Interest

The authors declare that the research was conducted in the absence of any commercial or financial relationships that could be construed as a potential conflict of interest.

## Publisher's Note

All claims expressed in this article are solely those of the authors and do not necessarily represent those of their affiliated organizations, or those of the publisher, the editors and the reviewers. Any product that may be evaluated in this article, or claim that may be made by its manufacturer, is not guaranteed or endorsed by the publisher.

## Supplementary Material

Supplementary Figure 1 Effects of Met deficiency in rearing period diets on the alpha diversity of the gut microbiota in egg-laying chicks at 6 weeks. **(A)** Sobs index of the community diversity. **(B)** Chao index of the community richness. **(C)** Shannon index of the community diversity. **(D)** Ace index of the community richness.
