# Supplementary figures and images for: Effect of Methionine Deficiency on the Growth Performance, Serum Amino Acids Concentrations, Gut Microbiota and Subsequent Laying Performance of Layer Chicks
Source: Front Vet Sci. 2022 Apr 25;9:878107. doi: 10.3389/fvets.2022.878107 (PMC9083200; doi:10.3389/fvets.2022.878107)

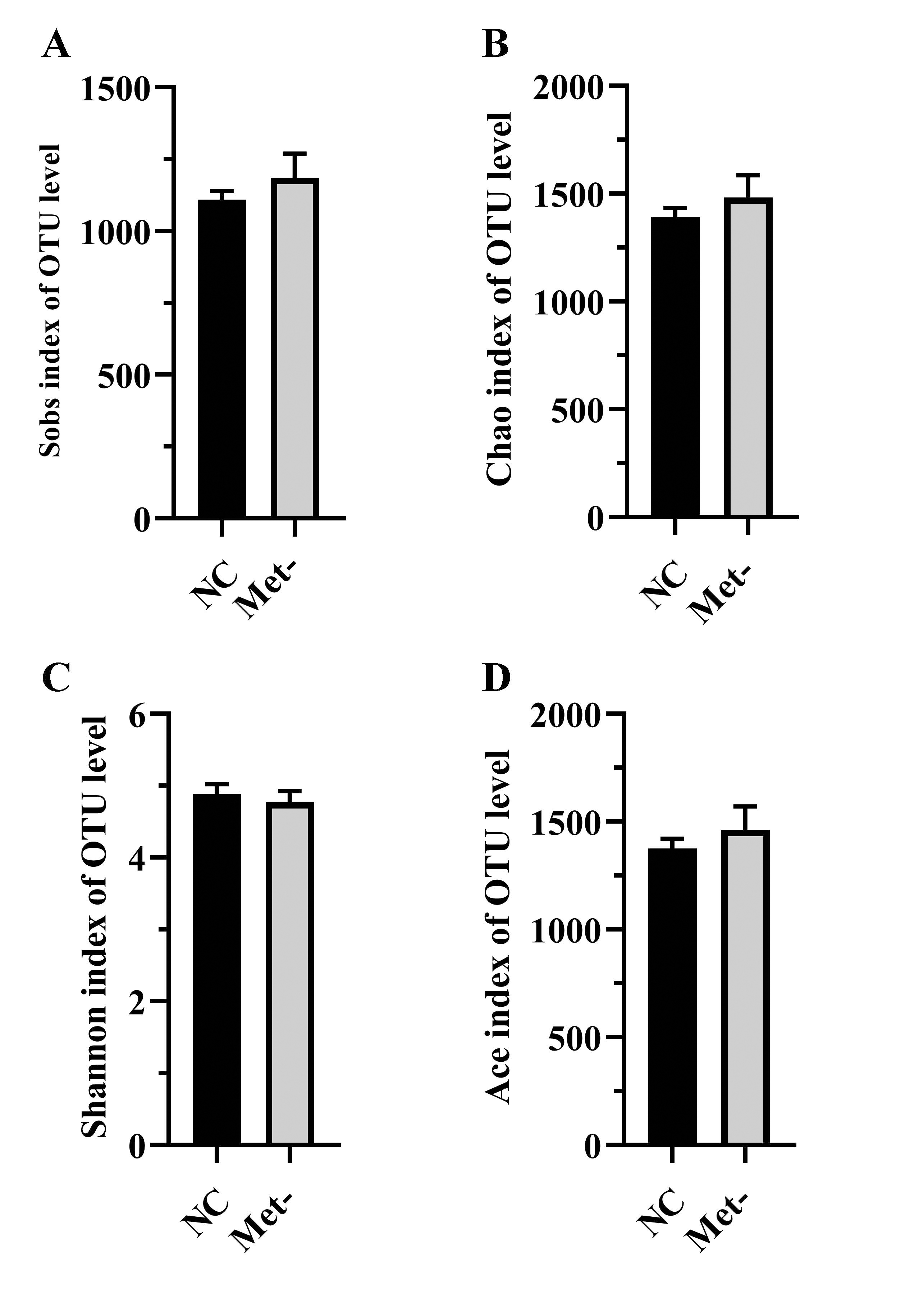

Supplement: Supplementary Figure 1 — Effects of Met deficiency in rearing period diets on the alpha diversity of the gut microbiota in egg-laying chicks at 6 weeks. (A) Sobs index of the community diversity. (B) Chao index of the community richness. (C) Shannon index of the community diversity. (D) Ace index of the community richness. [file Image_1.JPEG]
